# Berberine modulates expression of *mdr*1 gene product and the responses of digestive track cancer cells to Paclitaxel

**DOI:** 10.1038/sj.bjc.6690710

**Published:** 1999-10

**Authors:** H-L Lin, T-Y Liu, C-W Wu, C-W Chi

**Affiliations:** Departments of Medical Research and Education, Surgery, Veterans General Hospital-Taipei, Taiwan, ROC; Institute of Pharmacology, School of Medicine, National Yang-Ming University, Taipei, Taiwan, ROC

**Keywords:** oral, gastric and colon cancer cells, pgp-170, berberine, Paclitaxel

## Abstract

Berberine is the major constituent of *Coptis chinese* and is commonly used in Chinese herbal medicine to treat patients with gastrointestinal disorders. In this study, using flow cytometry, we have found that a 24-h berberine treatment up-regulated the multidrug-resistant transporter (pgp-170) expression in two oral (KB, OC2), two gastric (SC-M1, NUGC-3) and two colon (COLO 205, CT 26) cancer cell lines. Decreased retention of rhodamine 123 was observed in berberine-treated cells as compared to vehicle control. To examine whether the berberine modulated pgp-170 expression in cancer cells is associated with changes in drug resistance, we determined the cytotoxicity, cell cycle progression and cell morphology of Paclitaxel-treated cells. Paclitaxel (1 nM–10 μM) treatment for 24 h induced cytotoxicity in OC2, SC-M1 and COLO 205 cells in a dose-dependent manner. Pretreatment of cells with 32 μM berberine for 24 h prior to Paclitaxel treatment resulted in increased viability as compared to that of Paclitaxel-treated cells. In addition, Paclitaxel-induced apoptosis and/or G2/M arrest in these three cancer cell lines. Pretreatment of cells with berberine prior to Paclitaxel blocked the Paclitaxel-induced cell cycle responses and morphological changes. These results together suggest that berberine modulated the expression and function of pgp-170 that leads to reduced response to Paclitaxel in digestive track cancer cells. © 1999 Cancer Research Campaign


*British Journal of Cancer* **81**(3), 416–422
© 1999 Cancer Research Campaign
Article no. bjoc.1999.0710

### Berberine modulates expression of *mdr*1 gene product and the responses of digestive track cancer cells to Paclitaxel

**H-L Lin[1,3], T-Y Liu[1,3], C-W Wu[2,4] and C-W Chi[1,3]**

Departments of [1]Medical Research and Education and [2]Surgery, Veterans General Hospital-Taipei, Taiwan, ROC; [3]Institute of Pharmacology and [4]School of Medicine, National Yang-Ming University, Taipei, Taiwan, ROC

**Summary** Berberine is the major constituent of *Coptis chinese* and is commonly used in Chinese herbal medicine to treat patients with gastrointestinal disorders. In this study, using flow cytometry, we have found that a 24-h berberine treatment up-regulated the multidrug-resistant transporter (pgp-170) expression in two oral (KB, OC2), two gastric (SC-M1, NUGC-3) and two colon (COLO 205, CT 26) cancer cell lines. Decreased retention of rhodamine 123 was observed in berberine-treated cells as compared to vehicle control. To examine whether the berberine modulated pgp-170 expression in cancer cells is associated with changes in drug resistance, we determined the cytotoxicity, cell cycle progression and cell morphology of Paclitaxel-treated cells. Paclitaxel (1 nM–10 μM) treatment for 24 h induced cytotoxicity in OC2, SC-M1 and COLO 205 cells in a dose-dependent manner. Pretreatment of cells with 32 μM berberine for 24 h prior to Paclitaxel treatment resulted in increased viability as compared to that of Paclitaxel-treated cells. In addition, Paclitaxel-induced apoptosis and/or G2/M arrest in these three cancer cell lines. Pretreatment of cells with berberine prior to Paclitaxel blocked the Paclitaxel-induced cell cycle responses and morphological changes. These results together suggest that berberine modulated the expression and function of pgp-170 that leads to reduced response to Paclitaxel in digestive track cancer cells. © 1999 Cancer Research Campaign

**Keywords:** oral, gastric and colon cancer cells; pgp-170; berberine; Paclitaxel

Paclitaxel (taxol) isolated from the bark of Pacific yew tree *Taxus brovifolia*, had been reported to be effective against ovarian, breast and lung cancers in clinical trial (Chabner, 1991; Rowinsky and Donehower, 1991; Slichenmyer and Von Hoff, 1991; Anonymous, 1997; Belani, 1998). The underlying mechanism is enhancement of the tubulins and microtubules dynamic equilibrium and stabilized microtubules (Wilson, 1975; Dustin, 1980; Rowinsky et al, 1990). Thus, Paclitaxel-treated cells are arrested at G2/M phase (Milross et al, 1996). In addition, Paclitaxel-induced apoptosis and protease activation are common features of Paclitaxel-sensitive cells (Vikhanskaya et al, 1998).

It is well known that some cancer cells are more resistant to Paclitaxel treatment than others. Paclitaxel resistance can be separated into two types. One is a multidrug resistance (MDR)-related type (Dumontet et al, 1996) and the other is a non-MDR-related type. The human MDR1 gene coding for MDR transporter (pgp-170) belongs to ATP-binding cassette (ABC) transporter superfamily. The MDR1 overexpression has been reported in solid tumours, including colon cancer, renal carcinoma, hepatoma and pancreatic carcinoma (Goldstein, 1996). Uptake and/or efflux of isotope-labelled drugs or rhodamine 123 are frequently used for functional assay of pgp-170 in tumour cells. It has been found that the accumulation of chemotherapy agents, including Paclitaxel, or rhodamine 123 in tumour cells with overexpressed pgp-170 was significantly reduced as compared to the MDR antagonist-treated tumour cells (Watt et al, 1990; Chaudhary and Roninson, 1991; Kiehntopf et al, 1994). The other Paclitaxel resistance type includes TRAG-1–5 (Paclitaxel resistance-associated gene 1–5) (Duan et al, 1997), p53 expression (Catherine et al, 1995) (Vikhanskaya et al, 1998), alteration of β-tubulin isoform (Dumontet et al, 1996), changes in the level of intracellular glutathione (Liebmann et al, 1993), or cell growth morphology (Frankel et al, 1997).

Berberine, an alkaloid isolated from *Coptis chinese*, *Hydrastis canadensis* L., *Berberidaceae*, has been found to have antibacterial (Sun et al, 1988) and anti-inflammatory effects. Moreover, the *Coptis chinese* is frequently utilized in Chinese herbal medicine. The mechanism of berberine-induced growth inhibition in tumour cells is not well understood, but it has been documented that berberine interacts with DNA to form complex (Jenning and Ridler, 1983; Taira et al, 1994), inhibits DNA, RNA and protein synthesis in sarcoma S180 cells in vitro (Creasey, 1979). In addition, berberine has also been found to affect cell cycle progression. Berberine-treated HL-60 cells had an increased G2/M phase population (Kuo et al, 1995). In our previous studies, we have found that berberine decreased α-fetoprotein secretion (Chi et al, 1994) and up-regulated pgp-170 expression in hepatoma cells (Lin et al, 1999).

In this study we examined the role of MDR expression on oral, gastric and colon cancer cells in response to Paclitaxel treatment; we show that berberine up-regulated the MDR1 expression and enhanced the efflux ability of this transporter on six cancer cell lines. In addition, increased MDR1 expression is accompanied with reduction of Paclitaxel-induced cytotoxicity, cell cycle alterations, apoptosis and morphology changes in cancer cells. These

*Received 23 November 1998*
*Revised 9 April 1999*
*Accepted 20 April 1999*

*Correspondence to:* C-W Chi, Department of Medical Research and Education, Veterans General Hospital-Taipei, Shih-Pai, Taipei 11217, Taiwan, ROC

results suggest that MDR1 expression is associated with attenuation of Paclitaxel-induced response.

### MATERIALS AND METHODS

### Cell culture

Human oral cancer cell lines, OC2 (Wong et al, 1990) and KB (obtained from American Type Culture Collection, ATCC); human gastric carcinoma cell line, SC-M1 (Jin et al, 1987) and human colon cancer cell line, COLO 205 (obtained from ATCC) were cultured in RPMI-1640 (Gibco, Grand Island, NY, USA) medium supplemented with 10% heat-inactivated fetal bovine serum (Hyclone, Logan, UT, USA) and 0.01 mg $ml^{-1}$ gentamycin (Gibco). A murine colon cell line, CT26, was cultured in Dulbecco's modified Eagles medium (DMEM; Gibco) containing 10% heat-inactivated fetal bovine serum (Hyclone), 0.01 mg $ml^{-1}$ gentamycin, and 0.1 mM non-essential amino acid. Human gastric carcinoma cell line, NUGC-3 (Sekiguchi et al, 1978) was cultured in RPMI-1640 and DMEM in a 1:1 ratio supplemented with L-glutamine (2 mM; Gibco), kanamycin (100 μg $ml^{-1}$; Gibco) and 10% heat-inactivated fetal bovine serum. The cells were kept in a humidified incubator with 5% carbon dioxide and 95% air at 37°C.

### Drug treatment

Berberine (Sigma; B 3251, St Louis, MO, USA) and Paclitaxel (Biomol Research Labs, PA, USA) were dissolved in double distilled water and dimethylsulphoxide (DMSO) respectively. Six cell lines were treated with berberine (0.32–320 μM) for 24 h. For some experiments, cells were pretreated with berberine (32 μM) for 24 h, followed by replacement with fresh medium containing DMSO (0.1%) or various concentrations of Paclitaxel (0.001, 0.01, 0.1, 1, 10 μM) for an additional 24 h. After drug treatment, cells were harvested by TEG (0.125% trypsin, 0.05% EDTA, 0.05 g $ml^{-1}$ glucose) and used for the following assays.

### MDR1 transporter expression

The cell pellet ($1 \times 10^6$) was first resuspended in 100 μl phosphate-buffered saline (PBS), followed by incubation with 20 μl (12.5 μg $ml^{-1}$) anti-human MDR1 monoclonal antibody conjugated with phycoerythrin (PE) (Immunotech, UIC2 clone) for 15 min at room temperature. Non-specific binding was determined with an isotype control of mouse PE-conjugated IgG1. After incubation, cells were washed twice with PBS then resuspended in 500 μl PBS for flow cytometic analysis.

### Multidrug-resistance protein expression

The cell pellets ($1 \times 10^6$) were added with lysing buffer (0.5% Triton X-100, 0.2 μg $ml^{-1}$ $Na_2EDTA.2H_2O$, and 1% bovine serum albumin in PBS) and left on ice for 15 min. The mixture was then added with 100% methanol pre-cooled to –20°C and centrifuged at 300 ***g*** for 5 min. The supernatant was discarded and pellet was washed with PBS again. For primary antibody staining, the cell pellets were incubated with 20 μl mouse anti-MRP monoclonal antibody (1:50 dilution) (Chemicon International Inc., QCRL-3 clone) at 4°C for 30 min. Non-specific binding was determined using mouse IgG2a antibody. The cells were washed with PBS followed by incubation with goat anti-mouse antibody conjugated with fluorescein isothiocyanate (FITC) (1:30 dilution) (Organon Teknika Co.) at room temperature for 30 min. Finally, cells were washed with PBS and the multidrug resistance protein (MRP) expression was examined by flow cytometry.

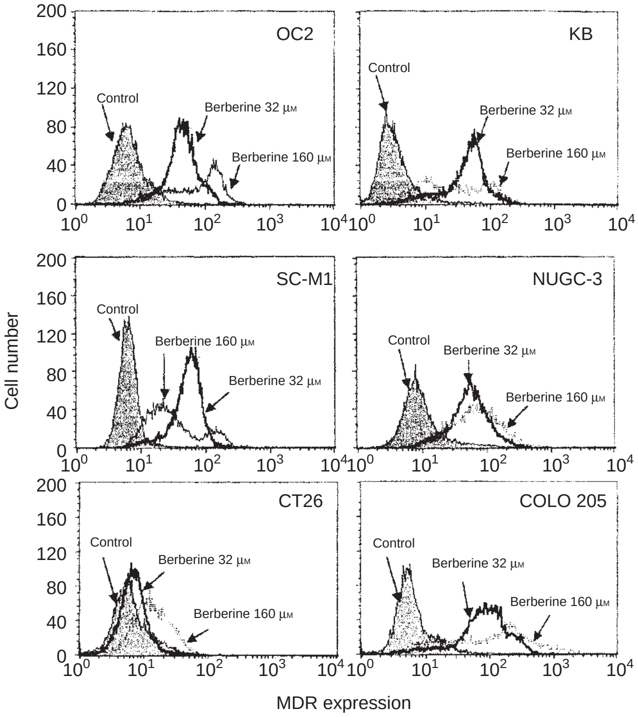

**Figure 1** The effect of berberine treatment on MDR1 expression was examined in two oral cancer cell lines (OC2, KB), two gastric cancer cell lines (SC-M1, NUGC-3) and two colon cancer cell lines (CT26, COLO 205). Cells were treated with indicated concentrations of berberine for 24 h followed by evaluation of MDR1 expression. MDR1 expression is estimated by flow cytometry. Data were acquired and analysed using FACS*Calibur*. A detailed description is in Materials and Methods

### Functional assay of MDR1 transporter

Previously, it has been reported that the retention of rhodamine 123 correlated well with the function of pgp-170 (Ludescher et al, 1992; Kiehntopf et al, 1994; Petriz and Garcia-Lopez, 1997; Salvioli et al, 1997). Therefore, retention of rhodamine 123 can be used as an indicator of pgp-170 function. Cells ($2–3 \times 10^5$) were treated with 0.32, 3.2, 32, 320 μM berberine for 24 h prior to the addition of 10 μg $ml^{-1}$ rhodamine 123 (Sigma). After incubation at 37°C for 5 min, cells were harvested and centrifuged at 300 ***g*** for 10 min. Cell pellets were resuspended with 500 μl PBS and immediately used for flow cytometic analysis of rhodamine 123 retention.

### MTT viability assay

Cells were cultured in 96-well cell culture cluster (Costar) at a density of $4 \times 10^4$ cells $ml^{-1}$. After drug treatment for 24–72 h, medium was discarded and replaced with an equal volume (100 μl) of fresh medium containing MTT (0.456 mg $ml^{-1}$; 3-[4,5-dimethylthiazol-2-yl]-2,5-diphenyl-tetrazolium bromide) for incubation 1.5 h in a 37°C incubator. The medium was discarded and then 100 μl DMSO were added. Cell viability was determined according to the colourimetric comparison by reading OD values

*© 1999 Cancer Research Campaign* 

**Table 1** The effect of berberine treatment on viability of OC2, SC-M1 and COLO 205 cells

| | Dose of berberine (μM) | | | |
|---|---|---|---|---|
| | 0.32 | 3.2 | 32 | 320 |
| OC2 | 105.3 ± 9.3 | 83.3 ± 3.9 | 75.2 ± 3.4 | 22.9 ± 7.3 |
| SC-M1 | 96.6 ± 8.2 | 90.0 ± 7.6 | 75.1 ± 9.2 | 17.8 ± 3.2 |
| COLO 205 | 97.2 ± 2.2 | 94.6 ± 5.1 | 72.3 ± 0.9 | 34.4 ± 3.2 |

Cells were treated with berberine or vehicle for 24 h and then used for MTT assay. Data (mean ± s.e.m.) represent the percentage of medium control from duplicate samples of three separate experiments.

from microplate reader (Spectra MAX 250) at absorption wavelength of 570 nm.

### Flow cytometric analysis of DNA content

The cell pellets ($1 \times 10^6$) were added with lysing buffer (0.5% Triton X-100, 0.2 μg $ml^{-1}$ $Na_2EDTA.2H_2O$ and 1% bovine serum albumin in PBS) and left on ice for 15 min. The mixture was then added with 100% methanol pre-cooled to –20°C and centrifuged at 300 ***g*** for 5 min. The supernatant was discarded and pellet was washed with PBS again. The pellet was stained with DNA staining solution (50 μg $ml^{-1}$-propidium iodide, 5 k units $ml^{-1}$ of RNaseA for 30 min at 4°C in the dark. The DNA content of each cell was measured using a Becton Dickinson FACS*Calibur* flow cytometer.

### Flow cytometry

Cells (10 000) were analysed on a Becton Dickinson FACS*Calibur* flow cytometer using an argon-ion laser (15 mWatt) with incident beam at 488 nm. Green fluorescence (rhodamine 123 or FITC) corresponding to efflux ability of MDR1 transporter or MRP expression were collected through a 530 nm filter, while the red fluorescence (PE) representing either the level of MDR1 transporter or DNA content (propidium iodide) was collected through a 585 nm filter. Data were acquired and analysed using FACS/CellQuest software on a Power Macintosh 7600/120 computer. Apoptotic cells and cells at specific cycle phases were determined by ModFit LT software.

### Morphology changes

To observe the cell morphology, OC2 and SC-M1 cells ($2 \times 10^6$ cells) were cultured on 75 $cm^2$ cell culture flasks (Corning) and treated with various doses of the drug. Photograph was taken at 300-fold magnification on an inverted phase-contrast microscope (Olympus; IMT-2)

### Statistical analysis

Statistical anslyses were performed using the Sigmastat software. Comparisons between the means of various treatment groups were analysed using Student's *t*-test or ANOVA. Tukey test was used for multiple comparisons. The difference was considered significant when $P < 0.05$.

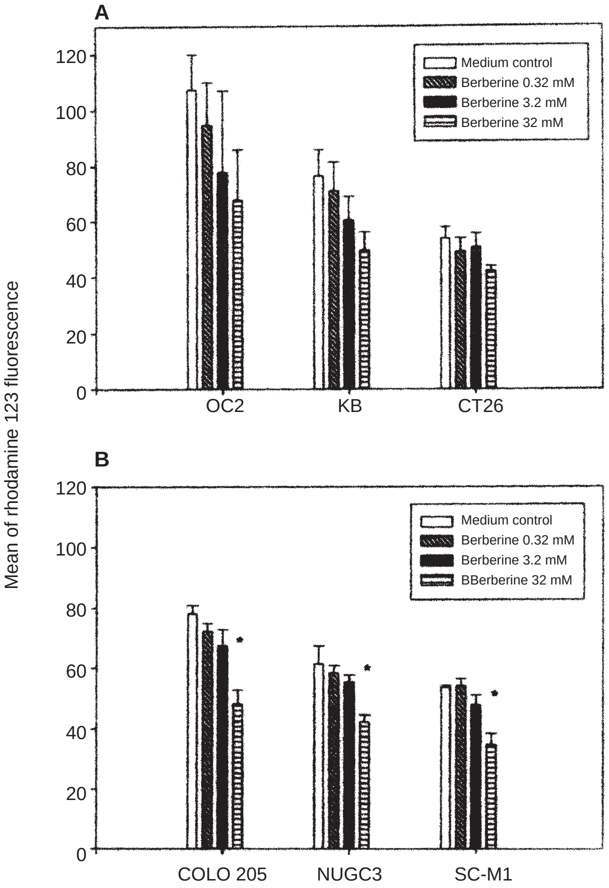

**Figure 2** The effect of berberine on rhodamine 123 retention in six digestive track cancer cell lines (OC2, KB, SC-M1, NUGC-3, CT26, COLO 205). Cells were treated with berberine (0.32 μM, 3.2 μM, 32 μM) for 24 h prior to the addition of rhodamine 123. (**A**) OC2, KB, CT26 cells (**B**) COLO 205, NUGC-3, SC-M1 cells. Data are the mean ± SE of duplicate samples from three independent experiments. *Indicates statistically significant difference ($P < 0.05$) as compared to the medium control group using Student's *t*-test

### RESULTS

Figure 1 shows the pgp-170 expression on berberine-treated six different cancer cell lines (OC2, KB, SC-M1, NUGC-3, CT26 and COLO 205). The expression of pgp-170 was increased significantly in five cell lines (OC2, KB, SC-M1, NUGC-3, COLO 205) after treatment with 32 μM berberine. No specific binding of pgp-170 was observed using isotype antibody. The expression of pgp-170 in CT26 cells was much less than the other five cell lines. When the berberine dose is increased to 160 μM or 320 μM, a further increase of pgp-170 expression was observed on OC2 and CT26 cells respectively. In comparison, no further increase in levels of pgp-170 was found on 160 μM berberine-treated KB, SC-M1, NUGC-3 and COLO 205 cells. Table 1 shows that 0.32 and 3.2 μM of berberine had little effect on cell viability. The viability of OC2, SC-M1 and COLO 205 cells decreased to 72.3–75.2% of control 24 h after a 32 μM berberine treatment. When the dose of berberine is increased to 320 μM, the viability further reduced to 17.8–34.4% of control. Since treatment of cells with 32 μM of berberine resulted in maximal expression of pgp-170 without significant effect on cell viability, we used 32 μM berberine treatment for further analyses.

© 1999 Cancer Research Campaign

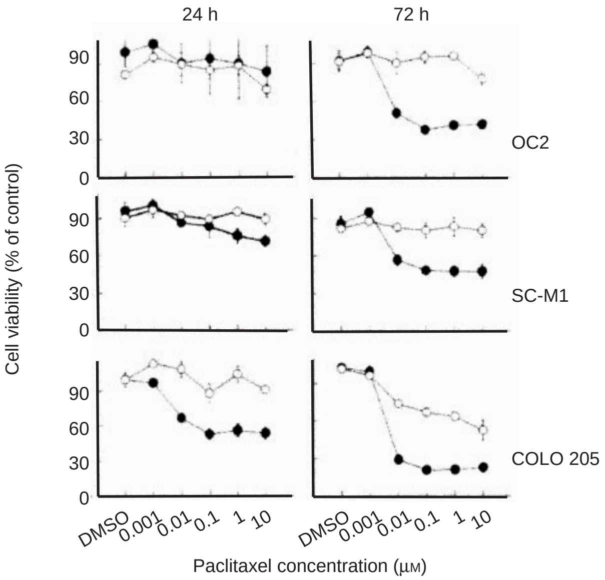

**Figure 3** The effect of Paclitaxel on cell viability of three cancer cell lines. Paclitaxel treated cells (solid circle) for 24 h or 72 h. Cells pretreated with 32 μM berberine for 24 h (open circle) and then treated with 1 nM–1 μM Paclitaxel or vehicle solvent, DMSO (0.1%), for an additional 24 or 72 h. Each point represents mean ± s.e.m. from duplicated samples of three separate experiments. *Indicates statistical difference at $P < 0.05$ using ANOVA analysis and Tukey test

It had been documented that the efflux of rhodamine 123 correlated well with pgp-170 expression. By this rationale, we used rhodamine 123 to evaluate whether the pgp-170 that expressed on cancer cells is functional. Figure 2 shows that two oral cancer cell lines (OC2, KB) had similar pattern of berberine-induced response. The efflux of rhodamine 123 showed a decreasing trend with the elevated dose of berberine. The efflux of rhodamine 123 in two gastric cancer cell lines (SC-M1, NUGC-3) and a colon cancer cell line (COLO 205) after 32 μM berberine treatment decreased significantly ($P < 0.05$) as compared with medium control. No significant decrease in rhodamine 123 retention was observed in berberine-treated CT26 cells. This corresponded well with our observation (Figure 1) that the pgp-170 expression was not increased in CT26 cells after a 32 μM berberine treatment.

In order to understand whether the enhanced efflux of pgp-170 correlates with drug resistance, we further investigated the effect of berberine treatment on Paclitaxel-induced response in three cell lines. Figure 3 shows that no significant decrease in viability of OC2, and SC-M1 cells was observed after Paclitaxel treatment for 24 h. The viability of COLO 205 cells showed a significant decrease ($P < 0.001$) after treatment with 10 nM–10 μM Paclitaxel for 24 h. No significant difference in viability was observed in cells treated with Paclitaxel alone and cells pretreated with berberine then treated with Paclitaxel ($P > 0.05$). When cancer cells were treated with 10 nM–10 μM Paclitaxel for 72 h, significant ($P < 0.05$) decreases in viability were observed in three cancer cell lines. Significantly higher viabilities ($P < 0.05$) were observed in berberine pretreatment groups than Paclitaxel (0.01–10 μM) treatment groups.

Previously, we had reported that low (10 nM) dose of Paclitaxel induced early apoptosis of SC-M1 cells while high (100 nM) dose Paclitaxel induced G2/M phase arrest prior to cell death (Chang et al, 1996). In order to determine whether berberine pretreatment affected Paclitaxel-induced cycle changes in cancer cells, cells were pretreated with berberine (32 μM) for 24 h followed by treatment with fresh medium containing low (10 nM) or high (100 nM) dose of Paclitaxel. Figure 4 shows that the DNA histogram of OC2 cells treated with berberine for 24 h (B 24, BW) had no remarkable alteration as compared with medium control (C24, C48). Low and high dose Paclitaxel treatment of OC2 cells resulted in apoptotic peaks and G2/M arrest respectively. In comparison, there were no significant changes on cell cycle in low (BL) or high (BH) dose Paclitaxel treatment of berberine-pretreated OC2 cells, as compared to vehicle controls (BD, BW). In SC-M1 cells, berberine treatment had no effect on cell cycle progression while low and high dose Paclitaxel treatment resulted in an apoptotic peak and a G2/M phase arrested cell population. These results were in accordance with our previous findings. However, it is

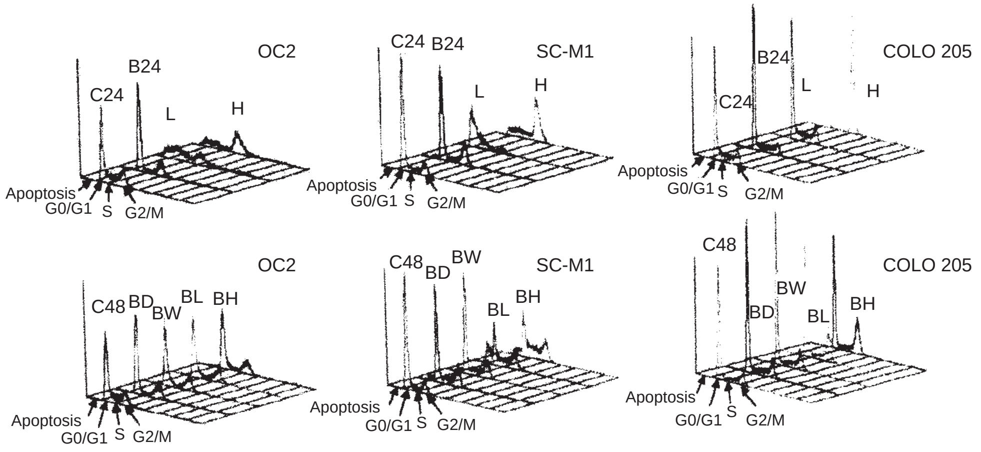

**Figure 4** The cell cycle progression on Paclitaxel-treated and berberine-pretreated cells. The cells were treated with low (L; 0.01 μM) or high (H; 0.1 μM) dose of Paclitaxel or berberine (B24; 32 μM) for 24 h as compared to the normal cell cycle profile (C24). To assess the effect of Paclitaxel on berberine-pretreated cells, cells were pretreated with berberine (32 μM) for 24 h followed by replacement with fresh medium (BW) containing DMSO (BD; 0.1%), low (BL; 0.01 μM) or high (BH; 0.1 μM) dose of Paclitaxel for another 24 h. C48 represents the cells that growth on normal condition for 48 h

*© 1999 Cancer Research Campaign*

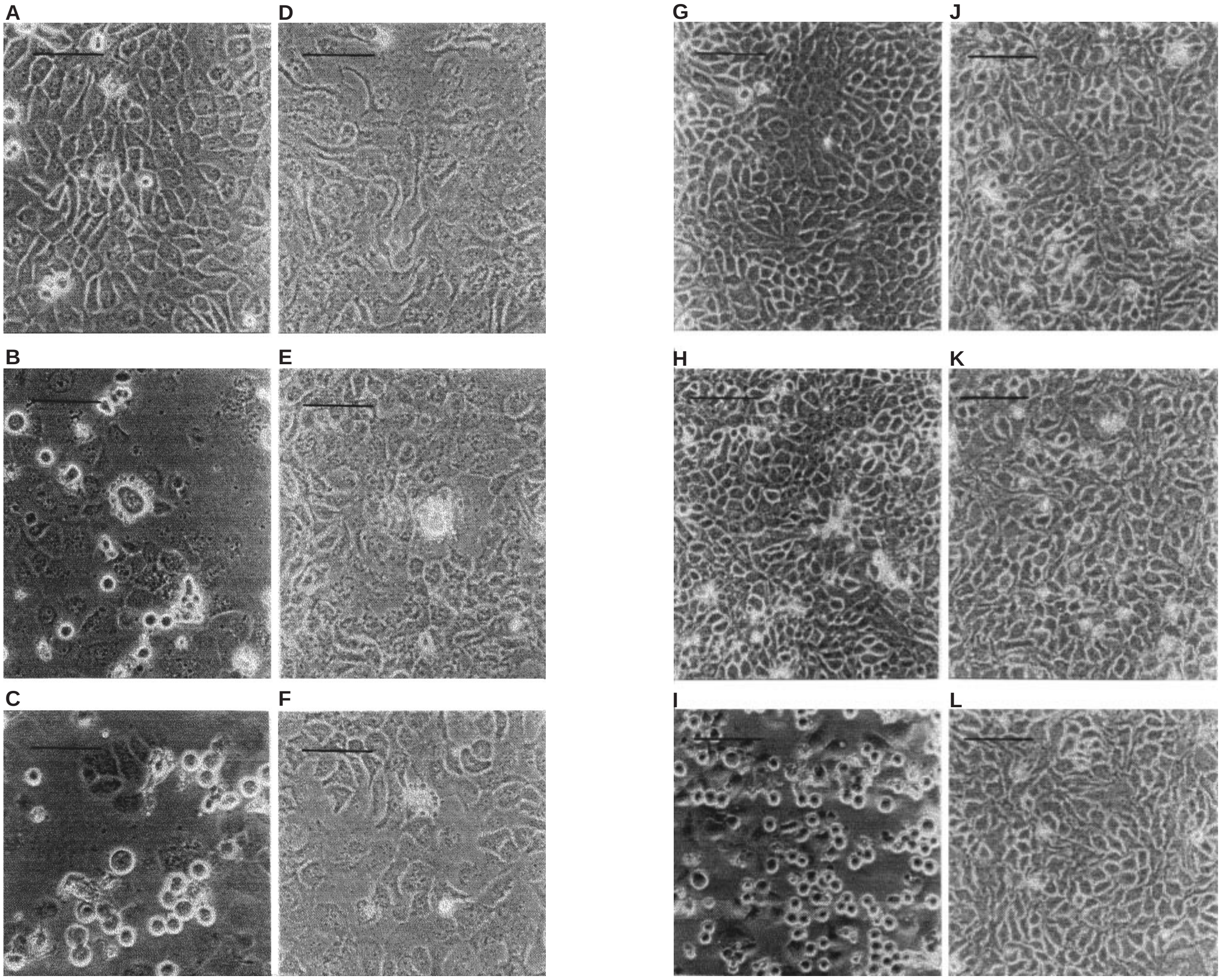

**Figure 5** The effect of Paclitaxel on cell morphology of OC2 (**A, B, C, D, E, F**) and SC-M1 (**G, H, I, J, K, L**). The cells were cultured on growth medium only (**A, G**) or treated with low (**B, H**; 0.01 μM) or high (**C, I**; 0.1 μM) Paclitaxel for 24 h. On the other hand, the cells were incubated with berberine (32 μM) for 24 h prior to replacement with fresh medium (**D, J**), low (**E, K**; 0.01 μM) or high (**F, L**; 0.1 μM) dose of Paclitaxel for additional 24 h. The microphotographs were taken with an inverted microscope. The bar length represent approximately 66 μM

interesting that pretreatment with berberine blocked the Paclitaxel-induced changes in cell cycle progression. No significant apoptosis and G2/M arrest was observed on low (BL) or high dose (BH) Paclitaxel treatment groups. For COLO 205 cells, high dose Paclitaxel treatment induced a significant G2/M phase arrest while low dose Paclitaxel treatment has no significant effect on cell cycle progression. Similarly, the Paclitaxel-induced cell cycle effect was blocked by berberine-pretreatment.

Figure 5 shows the effect of berberine on Paclitaxel-induced changes on morphology of OC2 (A-F) and SC-M1 cells (G-L). Low (10 nM) or high (100 nM) dose of Paclitaxel induced OC2 cells to round up or produce membrane blebbing. Similar morphological alterations were found on high (100 nM) dose Paclitaxel-treated SC-M1 cells. Berberine (32 μM) pretreatment for 24 h had no significant effect on both OC2 (Figure 5D) and SC-M1 cells (Figure 5J). In addition, pretreatment of OC2 and SC-M1 cells with berberine completely blocked the low or high dose Paclitaxel induced morphological changes.

In order to investigate whether MRP expression is also involved in berberine-induced effect, we further examined the MRP expression in six cancer cell lines treated with berberine for 24 h. A slight increase in the MRP expression was observed in berberine-treated cells. Figure 6 shows that a 32 μM berberine treatment for 24 h slightly increased the expression of MRP in OC2, SC-M1 and COLO 205 cell lines.

### DISCUSSION

Berberine had been documented to inhibit RNA, protein synthesis and integrate into DNA to form complex. Previously, synergistic cytotoxic effect of berberine (400 μM) and BCNU was found in brain tumour cells (Zhang et al, 1990), whether berberine has synergistic or antagonistic effect with other chemotherapeutic drug is not clear.

In this study, we have found that the cell growth of berberine (32 μM)-treated OC2, SC-M1 and COLO 205 cells was about 75% of medium control (Table 1). Berberine not only induced pgp-170 expression on six cancer cell lines (Figure 1) but also elevated the function of pgp-170 in these cancer cell lines (Figure 2). Nishino et al had found that berberine inhibited the effect of 12-*O*-tetradecanoylphorbol-13-acetate (TPA)-induced responses (Nishino et al, 1986). In another study, the accumulation of TPA and TPA-induced

*© 1999 Cancer Research Campaign*

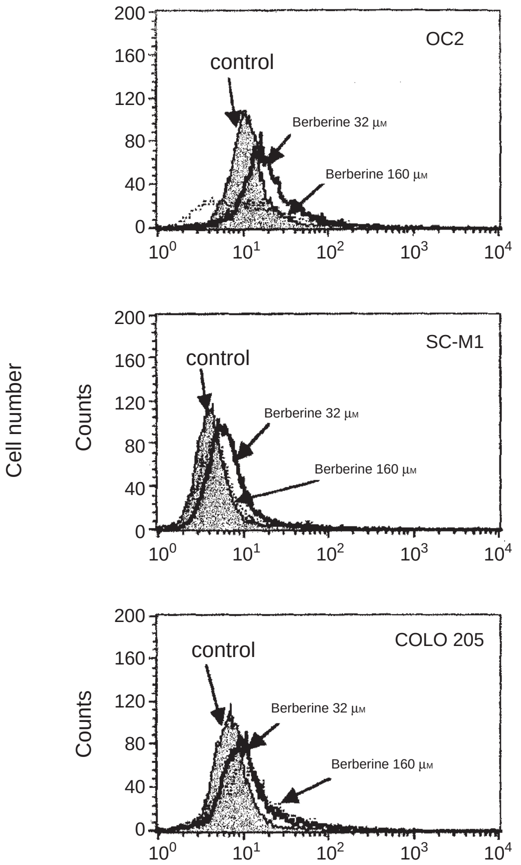

**Figure 6** The effect of berberine treatment on MRP expression in OC2, SC-M1 and COLO 205 cells. Cells were treated with different concentrations of berberine for 24 h followed by flow cytometric evaluation of MRP expression. Data were acquired and analysed using FACS*Calibur*. A detailed description is in Materials and Methods protein kinase C activity in MDR-expressed KB cells are slightly less than the MDR-negative KB cells (Cloud-Heflin et al, 1996). The decreased TPA-induced responses may be due to, at least in part, efflux of TPA by MDR transporter. Taken together, it seems reasonable to conclude that berberine may have modulated MDR1 expression and leads to reduced drug uptake in tumour cells.

Although the mechanism underlying berberine elevated pgp-170 expression is unclear but some reports indicated that cycloheximide-induced protein synthesis inhibition elevated pgp-170 mRNA expression in HepG2 cells (Gant et al, 1992; Schuetz et al, 1995). The cycloheximide-induced polysome stabilization may have contributed to the elevation of pgp-170 mRNA expression. Berberine has been reported to inhibit protein synthesis in sarcoma 180 cells (Creasey, 1979), whether berberine stabilized polysome and increased pgp-170 mRNA in these cancer cells is worthy of further investigation.

In order to evaluate whether berberine-modulated pgp-170 function can interfere with chemotherapeutic drug response in tumour cells, we assessed the Paclitaxel effect on cancer cells pretreated with berberine. Berberine decreased Paclitaxel-induced cytotoxicity in OC2, SC-M1, COLO 205 cells (Figure 3). In addition, berberine blocked high (0.1 μM) dose Paclitaxel-induced cell cycle arrest at G2/M phase in OC2, SC-M1, COLO 205 and abrogated low (0.01 μM) dose Paclitaxel-elicited sub-G0/G1 apoptosis in OC2, SC-M1 cells (Figure 4). Many studies documented that combination of some chemotherapeutic drugs with Paclitaxel decreased the response of Paclitaxel (Akutsu et al, 1995; Kano et al, 1996*a*, 1996*b*). Most of these drugs arrested tumour cells at S phase and the reduction of cells at G0/G1 or G2/M phases most likely contributed to reduced responses to Paclitaxel. In a previous study, we had reported that low dose of Paclitaxel induced apoptosis in gastric cancer cells (Chang et al, 1996) and this effect is cycle-dependent (Lin et al, 1998). It is interesting that similar effect was observed in OC2 cells. Berberine-induced cell cycle arrest at G2/M phase and apoptosis in Balb/c 3T3 cells (Yang et al, 1996), however, the dose of berberine used (268–537 μM) was much higher than we used (32 μM) in this study. In this study, berberine treatment did not induce G2/M arrest and berberine attenuated Paclitaxel-induced response was not the result of berberine-elicited cycle arrest effect (Figure 4).

Clinically, the phase II clinical trial of Paclitaxel had been performed on patients with oesophagus, gastric, hepatic or colon cancer (Rowinsky et al, 1992; Einzig et al, 1993; Ajani et al, 1994; Chao et al, 1998). The response rates were dependent upon cancer types and administered drug concentration, but many reports indicated that the low response rates were possibly associated with MDR1 expression. In this study, we have found that berberine up-regulated MDR1 expression in tumour cells (Figure 1) and the pgp-170 was functional (Figure 2). Most importantly, pretreatment of cancer cells with berberine blocked Paclitaxel-induced responses (Figures 3–5). These results suggest that MDR1 may be involved in low response rates of Paclitaxel. At present, no information is available on clinical combination of berberine and chemotherapeutic agents for treatment of cancer patients. However, it is worthy to point out that for patients receiving chemotherapy and simultaneously taking Chinese herbal medicine containing berberine, reduced therapeutic effect may result from up-regulation of MDR1.

Recently, another member of ABC family, MRP, had been found with high levels of MRP1, cMOAT, MRP3 or MRP5 expression in stomach, liver, colon or salivary gland respectively (Kool et al, 1997). In this study, we have found that berberine treatment slightly enhanced MRP expression in OC2, SC-M1 and COLO 205 cancer cells (Figure 6). It seems that the contribution of this slightly increased MRP in reduction of rhodamine 123 retention may be limited. Nevertheless, our results are the first to point out that there is antagonistic action between Paclitaxel and berberine mediated, at least in part, by MDR1 expression.

### ACKNOWLEDGEMENTS

We are grateful to Dr WC Lin (Institute of Biomedical Science, Academia Sinica, Taiwan) and Dr IJ Fidler (MD Anderson, Texas) for providing murine colon cancer cell line, CT 26. This study was supported in part by grants from National Science Council (NSC-87-2316-B075-002) and Veterans General Hospital-Taipei (VGH-86-390).

### REFERENCES

Ajani JA, Ilson D, Daugherty K, Pazdur R and Kelsen DP (1994) Taxol (Paclitaxel) is active against carcinoma of the esophagus: report of Phase II trial. *Proc Am Soc Clin Oncol* **13**: 192

Akutsu M, Kano Y, Tsunoda S, Suzuki K, Yazawa Y and Miura Y (1995) Schedule-dependent interaction between paclitaxel and doxorubicin in human cancer cell lines in vitro. *Eur J Cancer* **31A**: 2341–2346

© 1999 Cancer Research Campaign 

Anonymous (1997) Paclitaxel and docetaxel in breast and ovarian cancer. *Drug Ther Bull* **35**: 43–46

Belani CP (1998) Paclitaxel/carboplatin in the treatment of non-small-cell lung cancer. *Oncology* **12**: 74–79

Catherine MW, Jian Z, Patricia A, McQueney DB and Elias L (1995) Paclitaxel-induced mitotic block triggers rapid onset of a p53-independent apoptotic pathway. *Mol Med* **1**: 506–526

Chabner BA (1991) Paclitaxel. *Princ Pract Oncol* **5**: 1–10

Chang YF, Li LL, Wu CW, Liu TY, Lui WY, P'eng FK and Chi CW (1996) Paclitaxel-induced apoptosis in human gastric carcinoma cell lines. *Cancer* **77**: 14–18

Chao Y, Chan WK, Birkhofer MJ, Hu OP, Wang SS, Huang YS, Liu M, Whang-Peng, J, Chi KH, Lui WY and Lee SD (1998) Phase II and pharmocokinetic study of Paclitaxel therapy for unresectable hepatocellular carcinoma patients. *Br J Cancer* **78**: 34–39

Chaudhary PM and Roninson IB (1991) Expression and activity of P-glycoprotein, a multidrug efflux pump, in human hematopoietic stem cells. *Cell* **66**: 85–94

Chi CW, Chang YF, Chao TW, Chiang SH, P'eng FK, Lui WY and Liu TY (1994) Flowcytometric analysis of the effect of berberine on the expression of glucocorticoid receptors in human hepatoma HepG2 cells. *Life Sci* **45**: 2099–2107

Cloud-Heflin BA, McMaster RA, Osborn MT and Chamber TC (1996) Expression, subcellular distribution and response to phorbol esters of protein kinase C (PKC) isozymes in drug-sensitive and multidrug-resistant KB cells evidence for altered regulation of PKC-alpha. *Eur J Biochem* **239**: 796–804

Creasey WA (1979) Biochemical effects of berberine. *Biochem Pharm* **28**: 1081–1084

Duan Z, Feller A, Makastorsis T and Seiden MV (1997) Identification of a novel gene associated with Paclitaxel resistance in human cancer cells by differential display. *Proc Am Assoc Cancer Res* **38**: 482

Dumontet C, Duran GE, Steger KA, Beketic-Oreskovic L and Siki BI (1996) Resistance mechanisms in human sarcoma mutants derived by single-step exposure to Paclitaxel (Taxol). *Cancer Res* **56**: 1091–1097

Dustin P (1980) Microtubule. *Sci Am* **243**: 66–76

Einzig AI, Wiemik PH, Lipsitz S and Benson AB (1993) Phase II trial of Paclitaxel in patients with adenocarcinoma of the upper gastrointestinal track (UGIT): the Eastern Cooperation Oncology Group (ECOG) results. *Proc Am Soc Clin Oncol* **12**: 194

Frankel A, Buckman R and Kerbel RS (1997) Abrogation of Paclitaxel-induced G2-M arrest and apoptosis in human ovarian cancer cells grown as multicellular tumor spheroids. *Cancer Res* **57**: 2388–2393

Gant TW, Silverman JA and Thorgeirsson SS (1992) Regulation of P-glycoprotein gene expression in hepatocyte cultures and liver cell lines by a trans-acting transcriptional repressor. *Nucleic Acids Res* **20**: 2841–2846

Goldstein LJ (1996) MDR1 gene expression in solid tumor. *Eur J Cancer* **32**: 1039–1050

Jenning BR and Ridler PJ (1983) Interaction of chromosomal stains with DNA. An electrofluorescence study. *Biophys Struct Mech* **10**: 71–79

Jin L, Meng CL, Han SH, Ding MJ, Chang TM and Chen VTK (1987) A study on production of monoclonal antibodies using SC-M1 cells as immunogen. *J Med Sci* **8**: 17–25

Kano Y, Akutsu M, Tsunoda S, Ando J, Matsu J, Suzuki K, Inoue Y and Adachi KI (1996*a*) Schedule-dependent interaction between paclitaxel and 5-fluorouracil in human carcinoma cell lines in vitro. *Br J Cancer* **74**: 704–710

Kano Y, Akutsu M, Tsunoda S, Suzuki K and Yazawa Y (1996*b*) In vitro schedule-dependent interaction between paclitaxel and cisptatin in human carcinoma cell lines. *Cancer Chemother Pharmacol* **37**: 525–530

Kiehntopf M, Brach MA, Licht T, Petschauer S, Karawajew L, Kirschning C and Herrmann F (1994) Ribozyme-mediated cleavage of the MDR-1 transcript restores chemosensitivity in previously resistant cancer cells. *EMBO J* **13**: 4645–4652

Kool M, Haas MD, Scheffer GL, Scheper RJ, van Eijk MJT, Juijn JA, Baas F and Borst P (1997) Analysis of expression of cMOAT (MRP2), MRP3, MRP4, and MRP5, homologues of the multidrug resistance-associated protein gene (MRP1), in human cancer cell lines. *Cancer Res* **57**: 3537–3547

Kuo CL, Chou CC and Yung YM (1995) Berberine complex with DNA in the berberine-induced apoptosis in human leukemia HL-60 cells. *Cancer Lett* **93**: 193–200

Liebmann JE, Hahn SM, Cook JA, Lipschultz C, Mitchell JB and Kaufman DC (1993) Glutathione depletion by L-buthionine sulfoximine antagonized Paclitaxel cytotoxicity. *Cancer Res* **53**: 2066–2070

Lin HL, Chang YF, Liu TY, Wu CW and Chi CW (1998) Submicromolar Paclitaxel induces apoptosis in human gastric cancer cells at early G1 phase. *Anticancer Res* **18**: 3443–3450

Lin HL, Liu TY, Lui WY and Chi CW (1999) Up-regulation of multidrug resistance transporter expression by berberine in human and murine hepatoma cells. *Cancer* **85**: 1937–1942

Ludescher C, Thaler J, Drach D, Drach J, Spitaler M, Gattringer C, Huber H and Hofmann J (1992) Detection of activity of P-glycoprotein in human tumor samples using rhodamine 123. *Br J Haematol* **82**: 161–168

Milross CG, Mason KA, Hunter NR, Chung WK, Peter LJ and Milas L (1996) Relationship of mitotic arrest and apoptosis to antitumor effect of Paclitaxel. *J Natl Cancer Inst* **88**: 1308–1314

Nishino H, Kitagawa K, Fujiki H and Iwashima A (1986) Berberine sulfate inhibits tumor-promoting activity of teleocidin in two-stage carcinogenesis on mouse skin. *Oncology* **43**: 131–134

Petriz J and Garcia-Lopez J (1997) Flow cytometric analysis of P-glycoprotein function using rhodamine 123. *Leukemia* **11**: 1124–1130

Rowinsky EK, Cazenave LA and Donehower RC (1990) Paclitaxel: a novel investigational antimicrotubule agent. *J Natl Cancer Inst* **82**: 1247–1259

Rowinsky EK and Donehower RC (1991) Paclitaxel: twenty years later, the story unfold. *J Natl Cancer Inst* **83**: 1778–1781

Rowinsky EK, Onetto N, Canetta RM and Arbuck SG (1992) Paclitaxel: the first of the taxanes, an important new class of antitumor agents. *Semin Oncol* **19**: 646

Salvioli S, Ardizzoni A, Franceschi C and Cossarizza A (1997) JC-1, but not DiOC6(3) or rhodamine 123, is a reliable fluorescent probe to assess DY changes in intact cells: implications for studies on mitochondrial functionality during apoptosis. *FEBS Lett* **411**: 77–82

Schuetz JD, Strom SC and Schuetz EG (1995) Induction of P-glycoprotein mRNA by protein synthesis inhibition is not controlled by a transcriptional repressor protein in rat and human liver cells. *J Cell Physiol* **165**: 261–272

Sekiguchi M, Sakakibara K and Fuji G (1978) Establishment of cultured cell lines derived from a human gastric carcinoma. *Jpn J Exp Med* **48**: 61–68

Slichenmyer WJ and Von Hoff DD (1991) Paclitaxel: a new effective anti-cancer drug. *Anticancer Drug* **2**: 519–530

Sun D, Abraham SN and Beachey EH (1988) Influence of berberine sulfate on synthesis and expression of Pap fimbrial adhesion in uropathogenic *Escherichia coli*. *Antimicrob Agents Chemother* **32**: 1274–1277

Taira Z, Matsumoto M, Ishida S, Ichikawa T and Sakiya Y (1994) Aggregation of DNA enhanced by protoberberine alkaloids, coralyne and berberine. *Chem Pharm Bull* **42**: 1556–1561

Vikhanskaya F, Vignati S, Beccaglia P, Ottoboni C, Russo P, D'Incalci M and Broggini M (1998) Inactivation of p53 in a human ovarian cancer cell line increases the sensitivity to Paclitaxel by induction G2/M arrest and apoptosis. *Exp Cell Res* **241**: 96–101

Watt G, Long GW, Grogl M and Martin SK (1990) Reversal of drug-resistant falciparum malaria by calcium antagonists: potential for host cell toxicity. *Trans R Soc Trop Med Hyg* **84**: 187–190

Wilson L (1975) Microtubules as drug receptor: pharmacological properties of microtubule protein. *Ann NY Acad Sci* **253**: 213–231

Wong YK, Chang KW, Chen CF and Chang CS (1990) Characterization of 2 new cell lines derived from oral cavity human squamous cell carcinoma OC-1 and OC-2. *J Oral Maxillofac Surg* **48**: 385–390

Yang IW, Chou CC and Yung BY (1996) Dose-dependent effects of berberine on cell cycle pause and apoptosis in Balb/c 3T3 cells. *Naunyn-Schmiedebergs Arch Pharmacol* **354**: 102–108

Zhang RX, Dougherty DV and Rosenblum ML (1990) Laboratory studies of berberine used alone and in combination with 1,3-bis(2-chloroethyl)-1-nitrosourea to treat malignant brain tumors. *Chin Med J* **103**: 658–665

*© 1999 Cancer Research Campaign*
